# Outcomes of ST‐elevation myocardial infarction by age and sex in a low‐income urban community: The Montefiore STEMI Registry

**DOI:** 10.1002/clc.23412

**Published:** 2020-07-28

**Authors:** Anna E. Bortnick, Muhammad Shahid, Sanyog G. Shitole, Michael Park, Anna Broder, Carlos J. Rodriguez, James Scheuer, Robert Faillace, Jorge R. Kizer

**Affiliations:** ^1^ Department of Medicine, Division of Cardiology Montefiore Medical Center and Albert Einstein College of Medicine Bronx New York USA; ^2^ Cardiology Section, San Francisco Veterans Affairs Health Care System, and Department of Medicine University of California San Francisco San Francisco California USA; ^3^ Department of Medicine, Division of Cardiovascular Medicine University of Buffalo Buffalo New York USA; ^4^ Department of Medicine, Division of Rheumatology Montefiore Medical Center and Albert Einstein College of Medicine Bronx New York USA; ^5^ Department of Epidemiology and Biostatistics Albert Einstein College of Medicine Bronx New York USA; ^6^ NYC Health and Hospitals Jacobi Medical Center and North Central Bronx Hospital Bronx New York USA; ^7^ Department of Epidemiology and Biostatistics University of California San Francisco San Francisco California USA

**Keywords:** coronary artery disease, myocardial infarction

## Abstract

**Objectives:**

To compare outcomes by age and sex in race/ethnic minorities presenting with ST‐elevation myocardial infarction (STEMI), as studies are limited.

**Methods:**

We studied sociodemographics, management, and outcomes in 1208 STEMI patients evaluated for primary percutaneous coronary intervention between 2008 and 2014 at Montefiore Health System (Bronx, NY). A majority of patients self‐identified as nonwhite, and nearly two‐thirds were young (<45 years) or middle‐aged (45‐64 years).

**Results:**

Risk factors varied significantly across age groups; with more women and non‐Hispanic whites, hypertension, diabetes, dyslipidemia, prior cardiovascular disease, non‐sinus rhythm, and collagen vascular disease in the older age group (≥65 years); and higher body mass index, smoking, cocaine use, human immunodeficiency virus (HIV) infection and family history of heart disease in the young. Younger women had lower summary socioeconomic scores than younger men. Middle‐aged women had more obesity and dysmetabolism, while men had more heavy alcohol use. There was greater disease severity with increasing age; with higher cardiac biomarkers, 3‐vessel disease, cardiogenic shock, and coronary artery bypass grafting. Older patients had higher rates of death and death or readmission over 4.3 (interquartile range 2.4, 6.0) years of follow‐up. Middle‐aged women had higher rates of death or any readmission than men, but these differences were not significant after adjustment.

**Conclusions:**

These findings indicate a high burden of risk factors in younger adults with STEMI from an inner‐city community. Programs to target sociobehavioral factors in disadvantaged settings, including substance abuse, obesity, and risk of HIV, are necessary to more effectively address health disparities in STEMI and its adverse consequences.

## 1 INTRODUCTION

Over half of ST‐elevation myocardial infarction (STEMI) cases occur in adults <65 years old, with approximately 10% of all STEMIs affecting individuals <45 years of age.^1^, ^2^ Younger patients with acute STEMI are more often race/ethnic minorities as compared with their older counterparts.^1^ Among younger patients with acute myocardial infarction (AMI), inhospital mortality rates have been declining, but the same has not been the case for hospitalization rates.^3^ Younger women with AMI have been documented to have higher mortality than their male counterparts, in part related to differences in acute care.^4^ This heightened risk of mortality has been especially true for women race/ethnic minorities.^2^ Available studies, however, have mostly relied on administrative claims data, have often evaluated only short‐term outcomes, and have largely focused on non‐Hispanic whites and African Americans.^1^, ^4^, ^5^, ^6^, ^7^, ^8^, ^9^, ^10^

Here, we utilized a registry of patients presenting to a large inner‐city health system for acute care of STEMI to: (a) compare sociodemographic, behavioral, and clinical characteristics, patterns of care, and long‐term outcomes in young (<45 years), middle‐aged (45‐64 years), and older adults (≥65 years); and (b) to examine differences by sex in each of these age groups. We hypothesized that adverse social factors, manifest as unhealthful lifestyle habits and behaviors, would be overrepresented in younger adults with STEMI, particularly in women, and that these and corresponding differences in revascularization would account for higher event rates in the latter group.

## 2 METHODS

Patients presenting to Montefiore Health System (Bronx, NY) with acute STEMI from May 2008 to December 2014 were evaluated for inclusion in the Montefiore STEMI Registry, as previously described.^11^ Briefly, inclusion criteria were: symptoms concerning for an acute coronary syndrome lasting >20 minutes; evidence of ST‐segment elevation of ≥2 mm (≥1.5 mm for women) in leads V2‐3 or ≥ 1 mm in any other ≥2 contiguous leads or a new left bundle branch block; and elevation in troponin T (TnT) or creatinine kinase‐MB >99th percentile of the upper reference limit. Exclusion criteria were: inpatient status, end stage renal disease on hemodialysis, diabetic ketoacidosis, pregnancy, and in 2008 to 2009 but not thereafter, cardiogenic shock. Participants signed informed consent and the study was approved by the Institutional Review Board of the Albert Einstein College of Medicine. This study is a retrospective analysis of the Registry.

Clinical presentation, laboratory data, and procedural information on patients referred for cardiac catheterization for STEMI during the index hospitalization were abstracted by trained physicians and nurses. Additional clinical information and data on recurrent hospitalizations were obtained using Looking Glass Clinical Analytics (Streamline Health, Atlanta, Georgia), an interactive software application to search the electronic medical record.^12^ This was supplemented by direct review of records by trained physician abstractors. Cardiac catheterization data were obtained from an electronic database containing standardized angiographic and procedural information reported to New York State. Mortality data were obtained from the National Death Index. The North Bronx Health Network (NBHN), comprising two inner‐city safety net hospitals, is a common source of STEMI referrals to Montefiore and the NBHN electronic medical record was cross‐referenced for outcomes.

Race and ethnicity were self‐reported. Summary socioeconomic score (SSS), which relate to the wealth, income, education, and occupation of residents in a census‐block group (a proxy for neighborhood), was calculated as previously reported.^13^ Body mass index (BMI) was determined by weight (kg) divided by height squared (m^2^). Estimated glomerular filtration rate was derived from the Chronic Kidney Disease Epidemiology Collaboration equation.^14^ Hypertension was defined by history or use of antihypertensive medications. Diabetes was defined by history or use of antihyperglycemic agents.^15^ Hyperlipidemia was defined by history or use of an antihyperlipidemic drug. Current smoking was defined as use of ≥1 cigarette over the 30 days preceding the index event. Heavy alcohol intake was defined as >14 drinks/wk for men and >7 drinks/wk for women or a reported history of alcohol abuse. Previous cardiovascular disease (CVD) was defined as coronary heart disease (CHD), congestive heart failure (CHF), or stroke. Assessment of sinus rhythm was based on the initial electrocardiogram. Collagen vascular disease was based on International Classification of Diseases, Ninth Revision (ICD‐9) codes for systemic rheumatologic disorders and verified by rheumatologist review (A. Broder) of medical record. Family history of premature CHD was defined when such disease occurred in a first degree relative aged <55 (male) or <65 (female) years old. Human immunodeficiency virus (HIV) infection was defined as HIV seropositivity based on Western blot or detectable circulating viral RNA at any time point before or through the index STEMI hospitalization, as confirmed by linkage to the Montefiore Center for AIDS Research database. Critical coronary artery stenosis was defined by presence of ≥70% lesion in ≥1 coronary vessel or ≥50% in the left main coronary artery. Left ventricular ejection fraction (LVEF) was obtained either from ventriculography or transthoracic echocardiography. Myocardial infarction (MI) size was ascertained by peak TnT.^16^ Door‐to‐balloon time was identified as the period from hospital triage to balloon inflation in non‐transfer patients. Percutaneous coronary intervention (PCI) was defined per the National Cardiovascular Data Registry.^17^

Patients ranged in age from 24 to 96 years and were categorized into three groups: young (<45 years), middle‐aged (45‐64 years), and older (≥65 years) based on age at the index event. Baseline sociobehavioral, clinical, laboratory, angiographic, and acute care characteristics were compared among the three age groups and also by sex for each age group. Covariates were expressed as median (interquartile range [IQR]) for continuous variables and frequency and proportion for categorical variables. Comparisons of continuous variables applied the Wilcoxon rank sum test, while those of categorical variables used the chi‐square or Fisher's exact test, as appropriate.

Outcome measures were death, death or all‐cause readmission, and death or readmission for CVD. Readmission was defined as any hospital admission lasting ≥24 hours. Readmissions were limited to Montefiore and NBHN as retention to care is high.^18^ CVD hospitalizations were defined by discharge diagnosis, using ICD‐9 or Current Procedural Terminology, Fourth Edition, (CPT) codes consistent with coronary heart disease (angina pectoris, MI, PCI, coronary artery bypass grafting [CABG]), CHF, stroke, or atrial fibrillation. Follow‐up time was defined as time to the event of interest or through December 2015, whichever occurred earlier. Incidence rates were calculated per 100 person‐years and differences computed with Poisson 95% confidence intervals. Cox proportional hazards model was used for comparison of outcome between sexes within each age‐group. Adjustment was undertaken by covariates selected based on known or apparent associations with post‐STEMI outcomes. An initial model adjusted for age and race‐ethnicity with additional adjustment for BMI, SSS, hypertension (HTN), diabetes mellitus (DM), dyslipidemia, heavy alcohol use, history of collagen vascular disease, peak creatine phosphokinase (CPK), and 3‐vessel disease. The proportional hazards assumption was tested by Schoenfeld residuals, which revealed no violations. SAS version 9.4 (SAS Institute Inc., Cary, North Carolina) and GraphPad Prism 8 (La Jolla, California) were used. Statistical significance was set at *P* < .05 using a two‐sided test.

## 3 RESULTS

Of n = 1208 patients presenting with STEMI, nearly two‐thirds were young (<45 years) or middle‐aged (45‐64 years, Table 1). Women made up more than one‐third of the overall registry. The proportion of women experiencing STEMI increased with age; from roughly one‐fifth of the younger group, to over one‐fourth of the middle‐aged, to nearly one‐half of individuals in the older age group. In the overall registry, 37.1% self‐identified as Hispanic and 20.5% as non‐Hispanic black, 22.4% as non‐Hispanic white, and 20.0% were classified as other/unknown. “Other” included: American Indian or Alaska Native, Asian, Native Hawaiian or Other Pacific Islander, and multiracial. There were significantly fewer race/ethnic minorities across increasing age groups. The median SSS of STEMI patients was −2.16 (−5.27, −0.74), corresponding to ~2.2 SD below the national average. Median SSS did not vary by age or sex, but did vary by race/ethnicity (non‐Hispanic white −0.79 [−2.02, 0.22] vs Hispanic −3.67 [−6.42, −1.72] vs non‐Hispanic black −2.00 [−5.06, −1.02], *P* < .001).

**TABLE 1:** Baseline demographic, clinical, angiographic, and laboratory characteristics for young (<45 years), middle‐aged (45‐64 years), and older (≥65 years) adults presenting with STEMI

Characteristics	Young (n = 131)	Middle‐aged (n = 653)	Older (n = 424)	*P* for trend
Age, y	41 (37, 43)^a^ ^,^ ^b^	55 (50, 60)^a^ ^,^ ^c^	73 (69, 80)^c^ ^,^ ^b^	<.001
Men n (%)	105 (80.2)^b^	472 (72.3)^c^	233 (55.0)^c^ ^,^ ^b^	<.001
Race/ethnicity n (%)				
Non‐Hispanic white	22 (16.8)^b^	125 (19.1)^c^	123 (29.0)^c^ ^,^ ^b^	.001
Non‐Hispanic black	28 (21.4)	131 (20.1)	89 (21.0)	.955
Hispanic	51 (38.9)	241 (36.9)	156 (36.8)	.742
Other/unknown	30 (22.9)^b^	156 (23.9)^c^	56 (13.2)^c^ ^,^ ^b^	.001
Summary socioeconomic score	−2.35 (−5.47, −0.64)	−2.24 (−5.06, −0.75)	−2.05 (−5.44, −0.79)	.653
BMI, kg/m^2^	29.2^b^ (26.1, 32.2)	28.5^c^ (25.6, 32.4)	27.1^b^ (24.2, 30.2)	.001
SBP, mm Hg	131 (116, 149)	137 (117, 156)	136 (115, 153)	.451
DBP, mm Hg	83 (71, 96)^b^	82 (70, 94)^c^	74 (63, 88)^c^ ^,^ ^b^	<.001
Hypertension n (%)	51 (38.9)^a^ ^,^ ^b^	415 (63.6)^a^ ^,^ ^c^	341 (80.4)^c^ ^,^ ^b^	<.001
Diabetes mellitus n (%)	39 (29.8)	206 (31.6)^c^	160 (37.7)^c^	.030
Dyslipidemia n (%)	48 (36.6)^a^ ^,^ ^b^	350 (53.6)^a^ ^,^ ^c^	254 (59.9)^c^ ^,^ ^b^	<.001
Current smoking n (%)	76 (58.0)^a^ ^,^ ^b^	307 (47.2)^a^ ^,^ ^c^	79 (18.6)^c^ ^,^ ^b^	<.001
Cocaine use n (%)	17 (13.0)^a^ ^,^ ^b^	41 (6.3)^a^ ^,^ ^c^	5 (1.2)^c^ ^,^ ^b^	<.001
Heavy alcohol use n (%)	13 (9.9)	75 (11.5)^c^	32 (7.6)^c^	.129
Family history of CHD n (%)	50 (38.8)^b^	224 (34.7)^c^	89 (21.0)^c^ ^,^ ^b^	<.001
Prior CVD n (%)	18 (13.7)^a^ ^,^ ^b^	147 (22.5)^a^ ^,^ ^c^	137 (32.3)^c^ ^,^ ^b^	<.001
Collagen vascular disease n (%)	0 (0)	5 (0.8)^c^	10 (2.4)^c^	.013
HIV infection n (%)	7 (5.3)^b^	21 (3.2)^c^	2 (0.5)^c^ ^,^ ^b^	.001
Non‐sinus rhythm n (%)	7 (5.3)	39 (6.0)^c^	43 (10.1)^c^	.015
Killip class n (%)				
1	120 (91.6)^b^	575 (88.1)^c^	322 (75.9)^c^ ^,^ ^b^	<.001
2	6 (4.6)^b^	26 (4.0)	32 (7.6)^b^	.042
3	1 (0.8)^b^	10 (1.5)^c^	22 (5.2)^c^ ^,^ ^b^	.001
4	4 (3.1)^b^	42 (6.4)^c^	48 (11.3)^c^ ^,^ ^b^	.001
TIMI STEMI score	2 (1, 3)^b^	2 (1, 3)^c^	5 (4, 7)^c^ ^,^ ^b^	<.001
Peak troponin T, mg/mL	3.85 (2.14, 6.94)	4.43 (1.73, 8.30)	4.81 (2.15, 9.24)	.032
Peak CPK, U/L	1776 (8933251)^b^	1520 (688, 3270)^c^	1391 (618, 2650)^c^ ^,^ ^b^	.409
Initial creatinine, mg/dL	0.9 (0.7, 1.0)^a^ ^,^ ^b^	0.9 (0.8, 1.1)^a^ ^,^ ^c^	1.0 (0.8, 1.3)^c^ ^,^ ^b^	.001
Initial glucose, mg/dL	146 (118, 226)	152 (121, 210)^c^	162 (128, 238)^c^	.623
Door‐to‐balloon time, min	59 (43, 83)^b^	63 (45, 86)^c^	72 (50, 96)^c^ ^,^ ^b^	.362
CABG at index hospitalization n (%)	3 (2.3)	27 (4.1)	25 (5.9)	.064
Catheterization within 24 h n (%)	124 (95.0)	624 (95.6)	399 (94.1)	.535
Critically diseased vessels n (%)				
0	10 (8.1)^b^	44 (7.1)	48 (12.0)^b^	.027
1	74 (59.7)^a^ ^,^ ^b^	306 (49.0)^a^ ^,^ ^c^	153 (38.4)^c^ ^,^ ^b^	<.001
2	25 (20.2)^a^ ^,^ ^b^	183 (29.3)^a^	117 (29.3)^b^	.147
3	15 (12.1)^b^	91 (14.6)^c^	81 (20.3)^c^ ^,^ ^b^	.008
Culprit vessel n (%)				
LAD	60 (48.4)	286 (46.0)	183 (46.3)	.814
LCX	16 (12.9)	71 (11.4)	37 (9.4)	.228
RCA	48 (38.7)	265 (42.6)	175 (44.3)	.298
LVEF (%)	47 (39, 56)	50 (40, 59)^c^	47 (35, 59)^c^	.876
Discharge medications n (%)				
Aspirin	128 (97.7)	628 (99.2)	388 (98.7)	.826
Beta‐blocker	116 (88.6)^a^ ^,^ ^b^	593 (93.7)^a^	372 (94.7)^b^	.039
RAAS antagonist	100 (76.3)	481 (76.0)	280 (71.3)	.123
Statin	123 (93.9)	607 (95.9)	383 (97.5)	.068
Thienopyridine	125 (95.4)	607 (95.9)^c^	354 (90.1)^c^	.001

Abbreviations: BMI, body mass index; CABG, coronary artery bypass graft; CAD, coronary artery disease; CHD, coronary heart disease; CHF, congestive heart failure; CPK, creatine phosphokinase; CVD, cardiovascular disease; DBP, diastolic blood pressure; HIV, human immunodeficiency virus; LAD, left anterior descending; LCX, left circumflex; LVEF, left ventricular ejection fraction; RAAS, renin angiotensin aldosterone system; RCA, right coronary artery; SBP, systolic blood pressure; STEMI, ST‐elevation myocardial infarction; TIMI, thrombolysis in myocardial infarction.

a Young vs middle‐aged, *P* < .05.

b Young vs older, *P* < .05.

c Middle‐aged vs older, *P* < .05.

Risk factors and prevalent disease varied across age categories. BMI decreased with increasing age group. Younger patients were more likely to be current smokers or to use cocaine, as well as to have HIV infection or report a family history of CHD. By contrast, the prevalence of hypertension, diabetes, dyslipidemia, atherosclerotic CVD, CHF, non‐sinus rhythm, and collagen vascular disease increased in middle‐ and older‐age categories.

With regard to disease presentation and acute care, there was a significantly higher proportion of 3‐vessel coronary disease in older patients. Door‐to‐balloon time was shorter for young or middle‐aged vs older patients. The proportion of patients with higher Killip class at presentation increased across higher age groups. MI size, ascertained by peak TnT, was larger in older age groups, although LVEF was similar. Young individuals were less likely to be discharged on a beta‐blocker, while older adults were less likely to be discharged on a thienopyridine than young or middle‐aged adults.

There were differences in the pattern of sociodemographic and clinical factors between women and men in different age groups (Table 2). Women in the older age group had a higher age than men. Young and middle‐aged women were more impoverished than their male counterparts. Middle‐aged women had a higher BMI than men. Accordingly, they more frequently had hypertension, diabetes, and hyperglycemia on presentation as compared to men, although men were more likely to report heavy alcohol use. Young men more often had dyslipidemia as compared with women. Despite their worse risk profile, middle‐aged women had smaller infarcts, with lower cardiac biomarker concentrations and higher LVEF, than middle‐aged men. There were no significant differences in door‐to‐balloon time between men and women in any age group. The door‐to‐balloon time was shorter for young or middle‐aged vs older patients. Non‐system delays in older adults may be related to difficulty obtaining consent, atypical presentation, or altered mental status, which necessitates imaging to rule out a concomitant intracranial process. Additionally, older adults may have more complex coronary anatomy, which might make it more technically difficult to cross the culprit lesion and may be at higher risk of cardiac arrest or intubation preprocedure.^19^

**TABLE 2:** Baseline sociodemographic, clinical, angiographic, and laboratory characteristics for young (<45 years), middle‐aged (45‐64 years), and older (≥65 years) women vs men with acute STEMI

Characteristics	Young (n = 131)		Middle‐aged (n = 653)		Older (n = 424)	
	Women (n = 26)	Men (n = 105)	Women (n = 181)	Men (n = 472)	Women (n = 191)	Men (n = 233)
Age, y	40 (37, 42)	41 (38, 43)	56 (51, 60)	55 (50, 60)	75 (70, 82)	72 (68, 78)* ^,^ ** ^,^ ***
Race/ethnicity n (%)						
Non‐Hispanic white	4 (15.4)	18 (17.1)	33 (18.2)	92 (19.5)	52 (27.2)	71 (30.5)
Hispanic	8 (30.8)	43 (40.9)	69 (38.1)	172 (36.4)	71 (37.2)	85 (36.5)
Non‐Hispanic black	10 (38.5)	18 (17.1)	44 (24.3)	87 (18.4)	45 (23.6)	
Other/unknown	4 (15.4)	26 (24.8)	35 (19.3)	121 (25.6)	23 (12.0)	
Summary socioeconomic score	−3.5 (−7.0, −2.1)	−1.8 (−4.6, −0.5)* ^,^ **	−2.5 (−5.8, −1.1)	−2.1 (−4.2, −0.6)*	−2.0 (−5.1, −0.9)	−2.1 (−5.7, −0.7)
BMI, kg/m^2^	28.3 (25.9, 31.6)	29.2 (26.2, 32.2)	30.8 (26.8, 35.5)	28.1 (25.3, 31.3)* ^,^ ** ^,^ ***	28.1 (23.9, 31.2)	26.7 (24.2, 29.4)
SBP, mm Hg	131 (114, 142)	131 (117, 151)	136 (116, 154)	138 (118, 156)	132 (115, 151)	139 (116, 154)
DBP, mm Hg	77 (66, 93)	84 (71, 98)	79 (66, 91)	83 (70, 96)*	72 (60, 86)	76 (67, 89) *,**
Hypertension n (%)	10 (38.5)	41 (39.1)	127 (70.2)	288 (61.0)*	161 (84.3)	180 (77.3)
Diabetes mellitus n (%)	6 (23.1)	33 (31.4)	80 (44.2)	126 (26.7)* ^,^ ** ^,^ ***	71 (37.2)	89 (38.2)
Dyslipidemia n (%)	3 (11.5)	45 (42.9)* ^,^ **	107 (59.1)	243 (51.5)	122 (63.9)	132 (56.7)
Current smoking n (%)	13 (50.0)	63 (60.0)	89 (49.2)	218 (46.2)	30 (15.7)	49 (21.0)
Cocaine use n (%)	2 (7.7)	15 (14.3)	9 (5.0)	32 (6.9)	2 (1.1)	3 (1.3)
Heavy alcohol use n (%)	1 (3.9)	12 (11.4)	6 (3.3)	69 (14.6)* ^,^ ** ^,^ ***	3 (1.6)	29 (12.5)* ^,^ ** ^,^ ***
Family history of CHD n (%)	12 (46.2)	38 (36.9)	68 (37.8)	156 (33.5)	47 (24.6)	42 (18.0)
Prior CVD n (%)	4 (15.4)	14 (13.3)	43 (23.8)	104 (22.0)	54 (28.3)	83 (35.6)
Collagen vascular disease n (%)	0 (0.0)	0 (0.0)	3 (1.7)	2 (0.4)	8 (4.2)	2 (0.9)*
HIV n (%)	2 (7.7)	5 (4.8)	7 (3.9)	14 (3.0)	0 (0.0)	2 (0.9)
Non‐sinus rhythm n (%)	0 (0.0)	7 (6.7)	11 (6.1)	28 (5.9)	22 (11.5)	21 (9.0)
Killip class n (%)						
1	24 (92.3)	96 (91.4)	162 (89.5)	413 (87.5)	143 (74.9)	179 (76.8)
2	2 (7.7)	4 (3.8)	6 (3.3)	20 (4.2)	13 (6.8)	19 (8.2)
3	0 (0.0)	1 (1.0)	0 (0.0)	10 (2.1)	13 (6.8)	9 (3.9)
4	0 (0.0)	4 (3.8)	13 (7.2)	29 (6.1)	22 (11.5)	26 (11.2)
TIMI STEMI score	2 (1, 3)	2 (1, 3)	2 (1, 3)	2 (1, 3)	5 (4, 7)	5 (4, 6)* ^,^ **
Peak troponin T, mg/mL	3.1 (1.8, 7.0)	4.2 (2.3, 6.7)	3.2 (1.4, 6.7)	5.0 (1.9, 9.4)* ^,^ ** ^,^ ***	4.3 (2.2, 8.6)	5.3 (2.1, 10.3)
Peak CPK, U/L	1581(776, 2796)	1858 (916, 3251)	933 (520, 2068)	1779 (857, 3613)* ^,^ ** ^,^ ***	1178 (5532397)	1531 (691, 3000)*
Initial glucose, mg/dL	144 (111, 191)	150 (118, 235)	164 (127, 231)	149 (119, 201)*	164 (130, 239)	160 (125, 236)
Initial creatinine, mg/dL	0.7 (0.6, 0.8)	0.9 (0.8, 1.1)* ^,^ ** ^,^ ***	0.8 (0.7, 1.0)	1.0 (0.8, 1.2)* ^,^ ** ^,^ ***	0.9 (0.7, 1.2)	1.1 (0.9, 1.4)* ^,^ ** ^,^ ***
Door‐to‐balloon time, min	55 (43, 75)	60 (43, 86)	64 (50, 87)	63 (44,84)	72 (50, 103)	72 (47, 88)
CABG at index hospitalization, n (%)	2 (7.7)	1 (1.0)	6 (3.3)	21 (4.5)	13 (6.8)	12 (5.2)
Catheterization within 24 h n,%	24 (92.3)	100 (95.2)	175 (96.7)	449 (95.1)	182 (95.3)	217 (93.1)
Critically diseased vessels n (%)						
0	4 (16.7)	6 (6.0)	15 (8.6)	29 (6.5)	23 (12.6)	25 (11.5)
1	16 (66.7)	58 (58.0)	94 (53.7)	212 (47.2)	80 (44.0)	73 (33.6)
2	3 (12.5)	22 (22.0)	39 (22.3)	144 (32.1)	51 (28.0)	66 (30.4)
3	1 (4.2)	14 (14.0)	27 (15.4)	64 (14.3)	28 (15.4)	53 (24.4)
LVEF (%)	47 (39, 57)	48 (40, 56)	53 (43, 60)	50 (40, 58)* ^,^ **	49 (35, 60)	45 (35, 56)
eGFR, mL/min/1.73 m^2^	110 (90, 126)	107 (89, 116)	85 (67, 101)	89 (70, 101)	63 (45, 81)	68 (52, 84)
Discharge medications, n (%)						
Aspirin	25 (96.2)	103 (98.1)	174 (99.4)	454 (99.1)	177 (98.9)	211 (98.1)
Beta‐blocker	22 (84.6)	94 (89.5)	164 (93.7)	429 (93.7)	168 (93.9)	204 (94.9)
RAAS antagonist	17 (65.4)	83 (79.1)	130 (74.3)	351 (76.6)	124 (69.3)	156 (72.6)
Statin	24 (92.3)	99 (94.3)	170 (97.1)	437 (95.4)	173 (96.7)	210 (97.7)
Thienopyridine	24 (92.3)	101 (96.2)	168 (96.0)	439 (95.9)	160 (89.4)	194 (90.2)

Abbreviations: BMI, body mass index; CABG, coronary artery bypass graft; CAD, coronary artery disease; CHD, coronary heart disease; CVD, cardiovascular disease; CPK, creatine phosphokinase; DBP, diastolic blood pressure; HIV, human immunodeficiency virus; LVEF, left ventricular ejection fraction; RAAS, renin angiotensin aldosterone system; RCA, right coronary artery; SBP, systolic blood pressure; STEMI, ST‐elevation myocardial infarction; TIMI, thrombolysis in myocardial infarction.

* *P* < .05,

** *P* < .01,

*** *P* < .001.

**FIGURE 1:**
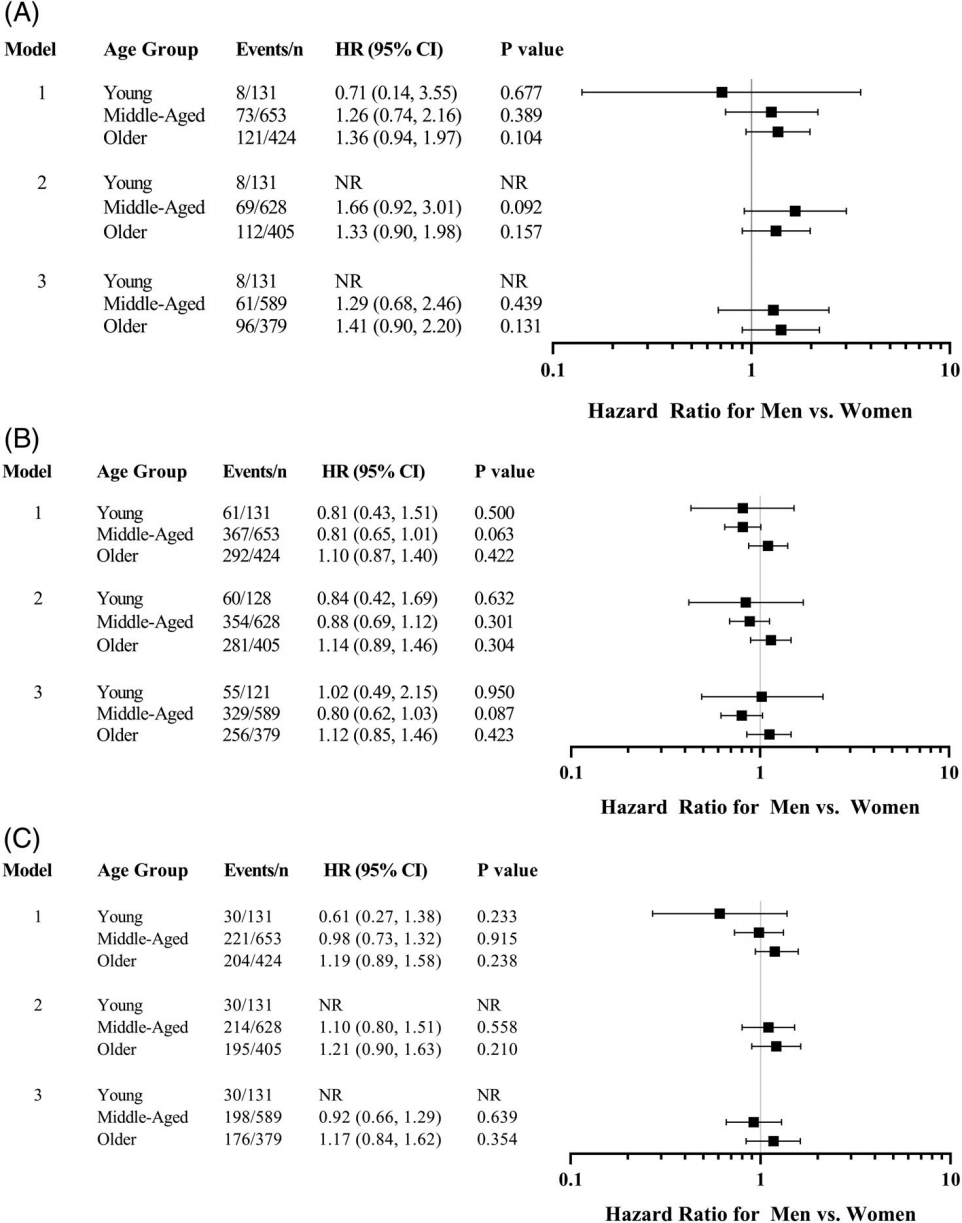
Hazard ratios and 95% confidence intervals for A, death; B, death or any readmission; and C, death or readmission for cardiovascular disease for men vs women (reference) across age groups. Model 1 adjusts for age and race/ethnicity; model 2 adjusts for model 1 variables, as well as body mass index, summary socioeconomic score, hypertension, diabetes mellitus, and dyslipidemia; and model 3 adjusts for model 2, heavy alcohol use, history of collagen vascular disease, peak creatine phosphokinase, 3‐vessel disease, and left ventricular ejection fraction. NR, not run because of small numbers

Over a median follow‐up time of 4.3 (IQR 2.4, 6.0) years, there was a significantly higher incidence of death in the older as compared to the younger‐age groups, as well as significantly greater rates of death or readmission (all‐cause or CVD) across increasing age groups (Table 3). Within specific age groups, a sex difference in outcomes was only observed in the middle‐aged category, where women had a higher incidence of death or readmission than men (Table 4). After adjustment for demographic and/or clinical risk factors, however, this higher risk for death or any readmission in middle‐aged women as compared to men ceased to be statistically significant (Figure 1A‐C,). Likewise, sex comparisons in the other age groups remained statistically nonsignificant after adjustment.

**TABLE 3:** Events and crude IRs of adverse outcomes for young (<45 years), middle‐aged (45‐64 years), and older (≥65 years) adults with acute ST‐elevation myocardial infarction

Outcomes	Entire Cohort	Young	Middle‐aged	Older	Young vs middle‐aged		Young vs older		Middle‐aged vs older	
					Difference (95% CI)	*P* value	Difference (95% CI)	*P* value	Difference (95% CI)	*P* value
Death										
Events n (%)	202 (16.7)	8 (6.1)	73 (11.2)	121 (28.5)		<.001		.082		<.001
IR^a^	4.4	1.4	2.7	9.2	1.3 (0.2, 2.5)	.062	7.8 (5.9, 9.7)	<.001	6.4 (4.7, 8.2)	<.001
Death or all‐cause readmission										
Events n (%)	720 (59.6)	61 (46.6)	367 (56.2)	292 (68.9)		<.001		.043		<.001
IR^a^	27.7	17.1	24.0	40.7	6.9 (2.0, 11.9)	.011	23.6 (17.2, 29.9)	<.001	16.6 (11.4, 21.9)	<.001
Death or CVD										
Events n (%)	455 (37.7)	30 (22.9)	221 (33.8)	204 (48.1)		<.001		.014		<.001
IR^a^	12.7	6.2	10.7	20.0	4.5 (1.9, 7.1)	.003	13.8 (10.3, 17.3)	<.001	9.3 (6.2, 12.4)	<.001

Abbreviations: CI, confidence interval; CVD, cardiovascular disease; IR, incidence rate.

a Per 100 patient‐years.

**TABLE 4:** Events and crude IRs of adverse outcomes per 100 person‐years for young (<45 years), middle‐aged (45‐64 years), and older (≥65 years) women vs men presenting with STEMI

Outcomes	Young				Middle‐Aged				Older			
	Men (n = 105)	Women (n = 26)	Difference^b^ (95% CI)	*P*	Men (n = 472)	Women (n = 181)	Difference^b^ (95% CI)	*P*	Men (n = 233)	Women (n = 191)	Difference^b^ (95% CI)	*P*
Events n (%)	6 (5.7)	2 (7.7)	—	—	55 (11.7)	18 (9.9)	—	—	71 (30.5)	50 (26.2)	—	—
IR^a^	1.3	2.1	−0.9 (−4.0, 2.3)	.529	2.9	2.4	0.5 (−0.9, 1.8)	.502	9.6	8.6	1.0 (−2.2, 4.2)	.555
Death or readmission												
Events n (%)	48 (45.7)	13 (50.0)	—	—	256 (54.2)	111 (61.3)	—	—	157 (67.4)	135 (70.7)	—	—
IR^a^	15.6	26.9	−11.4 (−26.7, 3.9)	.093	22.1	30.2	−8.1 (−14.4, −1.9)	.007	39.3	42.4	−3.1 (−12.5, 6.3)	.516
Death or CVD												
Events n (%)	22 (21.0)	8 (30.8)	—	—	159 (33.7)	62 (34.3)	—	—	116 (49.8)	88 (46.1)	—	—
IR^a^	5.3	11.1	−5.8 (−13.8, 2.2)	.092	10.5	11.1	−0.6 (−3.9, 2.6)	.683	20.9	18.9	1.9 (−3.5, 7.4)	.491

Abbreviations: CI, confidence interval; CVD, cardiovascular disease; IR, incidence rate; STEMI, ST‐elevation myocardial infarction.

a Per 100 patient‐years.

b Men minus women.

## 4 DISCUSSION

The present study examined long‐term adverse outcomes by age and sex categories in an acute STEMI registry from a US health system serving a disadvantaged urban population. There were four main findings of this investigation. First, younger patients (≤45 or 45‐64 years old) had a different risk profile than older patients (≥65 years old). Younger patients were more frequently men and nonwhite, had higher BMI, more frequent smoking, cocaine use, HIV infection, and family history of premature CHD. Older patients had prevalent hypertension, diabetes, dyslipidemia, prior CVD, non‐sinus rhythm, and collagen vascular disease. Second, younger women had a different risk profile than younger men. Younger women had a lower socioeconomic score than younger men, and notably, than all other age/sex groups. Women ≤45 years old less frequently had dyslipidemia than young men, but women 45‐64 years old had more obesity, hypertension and metabolic disease, though less heavy alcohol use, than their male counterparts. Third, the severity of STEMI was worse across rising age categories. There were higher cardiac biomarker levels and more frequent 3‐vessel disease, cardiogenic shock, and CABG during the index hospitalization, as well as higher beta‐blocker but lower thienopyridine use with increasing age. Last, there were increases in the rates of death and death or any readmission with increasing age. Outcomes were worse for middle‐aged women in comparison to men, but these differences were not significant after adjustment for covariates.

To our knowledge, the current investigation represents the first longer‐term assessment of post‐STEMI outcomes in a predominantly Hispanic and non‐Hispanic black sample from a low‐income urban setting. The higher burden of behavioral risk factors among younger adults documented here accords with a prior analysis of the American Heart Association's (AHA) Get with the Guidelines Coronary Artery Disease registry, where higher BMI and smoking were higher in patients <45 than in those ≥45 years old.^1^ Our registry provides additional information underscoring high‐risk behaviors in adults with premature STEMI, like substance abuse and HIV infection, which are independent risk factors for CHD.^20^, ^21^

As in the AHA Get with the Guidelines study, our registry also indicated a higher proportion of race/ethnic minorities among younger patients.^1^ There may be several reasons for this. Race/ethnic health disparities in cardiovascular risk factors and attendant disease are well recognized, contributing to overrepresentation of nonwhite patients in younger age categories.^22^, ^23^ Notably, the prevalence of diabetes in our young age group was almost 4‐fold that in the AHA Get with the Guidelines study, reflective of the epidemic of obesity and diabetes beginning earlier in childhood and adolescence, particularly among socially disadvantaged and race/ethnic minority groups.^24^ At the same time, because individuals from race/ethnic minorities may experience MI earlier than whites, they may not live to older age owing to cardiovascular sequelae or competing risks for death.^25^, ^26^ In addition, demographic trends specific to Bronx, NY, including immigration of younger adults from Latin America and to a lesser extent, Africa and Asia, have diversified the borough and the median age has become younger overtime.^24^, ^27^

Apart from added detail on behavioral risk factors, our registry also contains data on family history of premature CHD, delineating its association with younger age groups in our sample, with the <45‐year‐old group having nearly twice the prevalence of the ≥65‐year‐old group. This finding points to possible genetic or epigenetic contributions to acute STEMI and antecedent risk factors in our population.^28^ However, genetic influences have not been well studied in race/ethnic minorities, for which they may have greater or lesser impact in the context of other cardiovascular risk factors, like allostatic load, air pollution, constraints on physical activity, lower socioeconomic status, or tobacco smoke exposure.^24^, ^29^, ^30^

In our registry, young women had the lowest SSS of all the groups. Socioeconomic status is a foremost driver of race/ethnic health disparities, and a major determinant of behavioral and clinical risk factors, including smoking and other lifestyle habits, obesity, and diabetes.^24^, ^31^ In a combined analysis of two prospective registries, low socioeconomic status had the strongest association with mortality postacute MI independent of race, as compared with other risk factors.^32^ Apart from lower socioeconomic status, it is well documented that female adolescents have an increased risk of glycemic dysregulation as compared with their male counterparts, although in our study, significant differences were seen in the middle‐aged, but not young age group.^24^ That dyslipidemia was greater in young men than young women accords with the beneficial effects of estrogen on lipoprotein concentrations.^33^

The increasing long‐term risks of death and death or readmission observed with higher age categories are as expected. But there were notably high risks of death or CVD readmission, and especially, death or any readmission, for younger patients in our study. Patients <45 years old averaged a 17% annual incidence of death or any readmission during follow‐up, attesting to the high use of inhospital care post‐STEMI in this age group. A sex difference in death or any readmission, but not the other outcomes, emerged in the middle‐aged group, although this difference was not significant after adjustment for demographic and/or clinical factors. The high event rates observed in younger age groups, and by sex in the middle‐aged group, combined with the socioeconomic adversity, harmful habits, and high BMI documented in these groups, reinforce the importance of affordable and accessible community interventions for risk factor modification in low‐income settings, potentially tailored by sex and race/ethnicity.^34^, ^35^, ^36^

Sex‐based differences mostly for short‐term post‐MI outcomes have been documented in previous studies, particularly for younger women as compared with men.^1^, ^2^, ^4^, ^6^ Prior work has demonstrated longer door‐to‐balloon times, less evidence‐based medical therapy, and less primary PCI as potential factors contributing to a higher mortality rate for women vs men.^37^ Some studies also conclude that sex‐based differences in outcomes are attributable to the burden of comorbidities in women.^38^ However, in a large observational registry from New York state, extensive adjustment for comorbidities did not explain worse in‐hospital and 30 day mortality in women undergoing revascularization, particularly those middle‐aged and older. This suggests that there may be other contributing factors, like atypical presentation or delays in seeking medical attention which adversely impact STEMI outcomes in women.^39^ Nevertheless, protocolized STEMI care decreased disparities between men and women in a randomized controlled trial, offering a strategy to mitigate potential systemic biases in treatment.^40^ In our study, STEMI care was comparable between men and women and across all age groups, with comparable rates of catheterization and door‐to‐balloon times. Thus, a systems‐based approach should be multifaceted to reduce sex‐based disparities; by protocolizing STEMI care, targeting comorbid disease with sex‐specific, evidence‐based approaches among men and women across age groups, and educating the public to decrease delays in seeking medical attention.^39^, ^40^, ^41^, ^42^

Our study has several limitations. First, the study had a modest number of patients in the youngest age category, particularly for sex comparisons, limiting power to detect differences in risk profiles and outcomes. Second, this was an academic health system registry and results may not be generalizable to other populations. Third, there was no assessment of change in medical therapy overtime or use of cardiac rehabilitation, which may have influenced outcomes. Fourth, STEMI care is reflective of the time period spanning the registry and follow‐up.

In conclusion, the present study highlights important differences in sociodemographic, behavioral, and clinical risk factors between younger and older patients presenting with acute STEMI in a low‐income urban setting. Despite worse long‐term outcomes across increasing age groups, rates of death and readmission are substantial in younger age groups. In addition, the study shows a higher risk of death or readmission post‐STEMI in middle‐aged women as compared to middle‐aged men, findings that are partly explained by a higher burden of comorbidities in the former group. These findings emphasize the need for targeted community‐based interventions that improve lifestyle and clinical risk factors and are tailored to various race/ethnic, age groups, and sexes, in order to reduce the burden of STEMI and its associated complications in vulnerable US populations.

## CONFLICT OF INTEREST

Jorge R. Kizer reports stock ownership in Amgen, Bristol‐Myers Squibb, Gilead Sciences, Johnson & Johnson, Medtronic, Merck, and Pfizer. Anna E. Bortnick served as site principal investigator for multi‐center trials sponsored by Abbott, AstraZeneca, Sanofi‐Aventis, CSL‐Behring, for which her institution received compensation and received an honorarium from ClearView Healthcare Partners, LLC.
